# The Effect of Pessary Insertion on Pelvic Anatomy: A Prospective Observational Study Using Upright MRI

**DOI:** 10.1111/1471-0528.70280

**Published:** 2026-06-15

**Authors:** Annemarie van der Steen, Jaimy A. Simmering, Manon Perik, Frank F. J. Simonis, Frieda van den Noort, Anique T. M. Grob

**Affiliations:** ^1^ Department of Gynecology Ziekenhuisgroep Twente Hengelo the Netherlands; ^2^ Multi‐Modality Medical Imaging (M3i) Group, Technical Medical Centre University of Twente Enschede the Netherlands; ^3^ Magnetic Detection and Imaging (MD&I), TechMed Centre University of Twente Enschede the Netherlands

**Keywords:** magnetic resonance imaging, pelvic inclination correction system, pelvic organ prolapse, pessary, treatment, upright

## Abstract

**Objective:**

To determine the anatomical effect of pessaries in the upright position in women with pelvic organ prolapse (POP).

**Design:**

Prospective observational cohort study.

**Setting:**

Outpatient clinic of our gynaecology department.

**Population:**

Fifteen POP patients with successful ring‐shaped pessary use.

**Methods:**

Upright magnetic resonance (MR) images of the pelvis were acquired before, immediately after insertion of their pessaries, and 1 week later.

**Main Outcome Measures:**

Distances from the lowest points of the bladder, cervix, and pessary to the pelvic inclination correction system (PICS) line were measured, as well as the angles of the uterus and pessary relative to the PICS in the midsagittal plane.

**Results:**

Pessary insertion lifted the bladder's lowest point from 0.1 cm below to 0.6 cm above the PICS (*p* = 0.015), but it descended to 0.2 cm above the PICS 1 week later (*p* = 0.334). The cervix was lifted from 0.0 to 2.5 cm above the PICS (*p* < 0.001), which persisted after 1 week (*p* = 0.470). The angle between uterus and PICS increased after insertion from 97^o^ to 115^o^ (*p* = 0.008) and remained stable (113°, *p* = 0.198). Pessary position changed from 0.7 to 0.3 cm below the PICS in 1 week (*p* = 0.020); the angle did not change (56° to 54°, *p* = 0.820).

**Conclusion:**

Pessary insertion changes the pelvic organ position and angulation. After an initial lift, the effect on the bladder diminished after 1 week, while for the uterus it remained. This may suggest a role for uterus lift in relieving POP symptoms.

**Abbreviations**
CCMO: Centrale commissie mensgebonden onderzoek
IQR: Interquartile range
LAM: levator ani muscle
MR: Magnetic resonance
MRI: Magnetic resonance imaging
PICS: Pelvic Inclination Correction System
POP: Pelvic organ prolapse
POP‐Q: Pelvic organ prolapse quantification

## 1 Introduction

Pelvic organ prolapse (POP) has a prevalence of 11.4% in women between 45 and 85 years [1]. A pessary is a relatively accessible and inexpensive conservative treatment option that reduces POP symptoms. However, the success rates of pessary fitting range between 58% and 73% for the regular ring pessary [2, 3, 4, 5]. One of the main reasons to discontinue pessary treatment is dislodgement of the pessary. Earlier research showed that the main support mechanisms are the levator ani muscle (LAM) and the uterus [6]. Other reasons to discontinue pessary use are failure to relieve symptoms or side effects such as vaginal discharge [7, 8]. Therefore, to improve pessary design, not only the knowledge and understanding of the support mechanism for a pessary is important, but also its effect on the pelvic anatomy is paramount. Imaging can be used to obtain this knowledge and provide new insights into the success and failure of pessary treatment by evaluating the position of the pessary and pelvic organs. Magnetic resonance imaging (MRI) has already been proven insightful in relation to POP and pessary fitting. For example, POP is associated with LAM damage and pessaries that get dislodged generally lie lower in the pelvis and are more mobile during straining manoeuvres [9, 10]. MRI in upright position for POP assessment may even provide more insights, since it allows for imaging in the position in which POP is most prevalent and provides the most accurate insights in the interaction between the pessary and the pelvic organs. Previous studies using upright MRI focused on pelvic anatomy in POP patients, as well as the pessary support mechanisms, and those results suggest that residual bladder prolapse may still be present in women that are content with their pessary [6, 11]. This raises the question what anatomical effect a pessary should have, in order to relieve POP symptoms.

Therefore, the aim of this study was to evaluate the effect of successful pessary use on the pelvic organs in women with POP. By understanding pessary fit, a next step could be made towards the development of personalized pessaries for patients in whom conventional pessary treatment fails.

## 2 Methods

### 2.1 Patient Population

This prospective study is part of the “Evaluation of pessary placement on the anatomy of the pelvic organs in patients with pelvic organ prolapse using imaging techniques” (EPPA) umbrella study that was approved by the institution's board of directors and medical ethics committee CMO region Arnhem‐Nijmegen, The Netherlands and registered as NL74061.091.20. The current sub study included women with symptomatic POP and stage ≥ 2 on pelvic organ prolapse quantification (POP‐Q) of their anterior and/or apical compartment between February 2023 and June 2024 from the outpatient clinic of our gynaecology department. Additional inclusion criteria were age ≥ 18 years and satisfactory ring pessary (with or without support membrane) usage for at least 3 months with the intent to continue use. Patients were excluded if they were not able to stand for 20 min without assistance, were not eligible for MRI according to the MRI safety checklist, or waist circumference ≥ 143 cm or weight > 200 kg, because of the limited MRI coil circumference and table strength. The study was conducted according to the relevant guidelines and regulations. All patients gave written informed consent. All patients filled out a self‐developed questionnaire regarding their general health, pelvic floor symptoms and pessary usage (see supplementary file), and underwent upright MRI examinations.

### 2.2 MRI Examination

The patients removed their pessary or had their pessary removed 24 h prior to the first MRI scan, in order to let the prolapsed organs descend to their maximum extent. The time interval of 24 h was based on a previous study [12], in which most patients showed an immediate descent of their prolapse after pessary removal, but in 33% and 17% of patients this took up to 8 h for the bladder and cervix, respectively. Although in theory the organs could descend more if the time interval were even longer, due to ethical constraints, we decided not to remove the pessary more than 24 h before the first scan. MRI scans of the patients were obtained [1] prior to pessary re‐insertion [2], immediately after pessary re‐insertion and [3] 1 week after pessary re‐insertion [13]. Patients were instructed not to remove the pessary in the meantime. The patients were not allowed to drink for 1 h before the scan and had to empty their bladder within 15 min before the scan. The patients were standing with the pelvis at rest in a 0.25 T MR scanner (G‐Scan Brio; Esaote S.p.A., Genoa, Italy) tilted to 81° and imaged with a dedicated multichannel spine coil. The scan sequence is the same as used in previous research [12].

### 2.3 Image Analysis

Image analysis was performed as described by Van der Steen et al. [12] using 3D Slicer (v5.2.1, https://www.slicer.org) [14]. The extent of POP was evaluated on the upright MRI data using the Pelvic Inclination Correction System (PICS) reference line. The MRI PICS line was drawn in the midsagittal plane, where the Sacrococcygeal Inferior Pubic Point (SCIPP) line was rotated clockwise for 29 degrees to determine the upright PICS line, which was proven to provide a reference line perpendicular to the body axis for accurate measurements [15, 16, 17]. Next, the lowest points of the bladder, uterus and pessary were annotated manually and the perpendicular distances (d) between these points and the PICS line were calculated, where a positive value indicates a position below the PICS line and a negative value a position above the PICS line. A lift of the lowest point of ≥ 1 cm was deemed clinically relevant [18, 19]. In addition, the angle between the pessary and the PICS (pessary angle, a_Pessary_), and between the endometrium in the uterus and the PICS (uterus angle, a_Uterus_) were obtained (Figure S1).

### 2.4 Statistical Analysis

Statistical analyses were performed using SPSS version 28.0.1.0 (IBM SPSS Statistics, Chicago, IL, USA). Due to the explorative design of the study a sample size calculation was not feasible, and medical ethical approval was obtained for including 15 women, as a balance between patient burden and sufficient sample size to be able to draw conclusions. Normality of the data was assessed by visual inspection of histograms. Data were presented as medians (interquartile range, IQR). Differences in the pelvic organ and pessary position and orientation between the different scans were investigated using Friedman tests with post hoc Wilcoxon rank tests. A *p*‐value of < 0.05 was considered statistically significant.

No Artificial Intelligence Generated Content (AIGC) tools were used in the development of this manuscript.

## 3 Results

### 3.1 Demographics

A total of 15 patients were included in this study. An overview of the patients' demographics can be found in Table 1. All women still had their uterus and all women used a ring‐shaped pessary, of whom seven with and eight without support. In one woman, the uterus position and uterus angle could not be determined on the scan 1 week after pessary insertion because of poor image quality.

**TABLE 1:** Demographic data of the study population.

Age	65 [52–69]
BMI	23 [21–26]
Parity	2 [2–3]
Vaginal deliveries	2 [2–3]
Years since last delivery	27 [13–34]
Pessary size (mm)	76 [70–76]
POP‐Q bladder (cm)	1 [−1–2]
POP‐Q cervix (cm)	−2 [−4–3]

*Note:* Data is presented as median (interquartile range).

Abbreviation: BMI, body mass index.

### 3.2 Bladder Position

Immediately after pessary re‐insertion, the bladder, measured by its lowest point (median (IQR)), was on average lifted from 0.1 cm (−1.0 cm, 3.3 cm) below the PICS to −0.6 cm (1.0 cm, 1.4 cm) above the PICS (*p* = 0.015). 1 week later, the lowest point of the bladder (median (IQR)) descended to −0.2 cm (−1.0 cm, 3.1 cm) above the PICS (*p* = 0.334). Figure 1 shows the course of the lowest point of the bladder of individual patients, revealing variability in the bladder position among the cohort. In only three out of 15 patients (20%) did the lowest point of the bladder 1 week after pessary re‐insertion show a clinically relevant lift of 1 cm or more compared to prior to pessary insertion.

**FIGURE 1:**
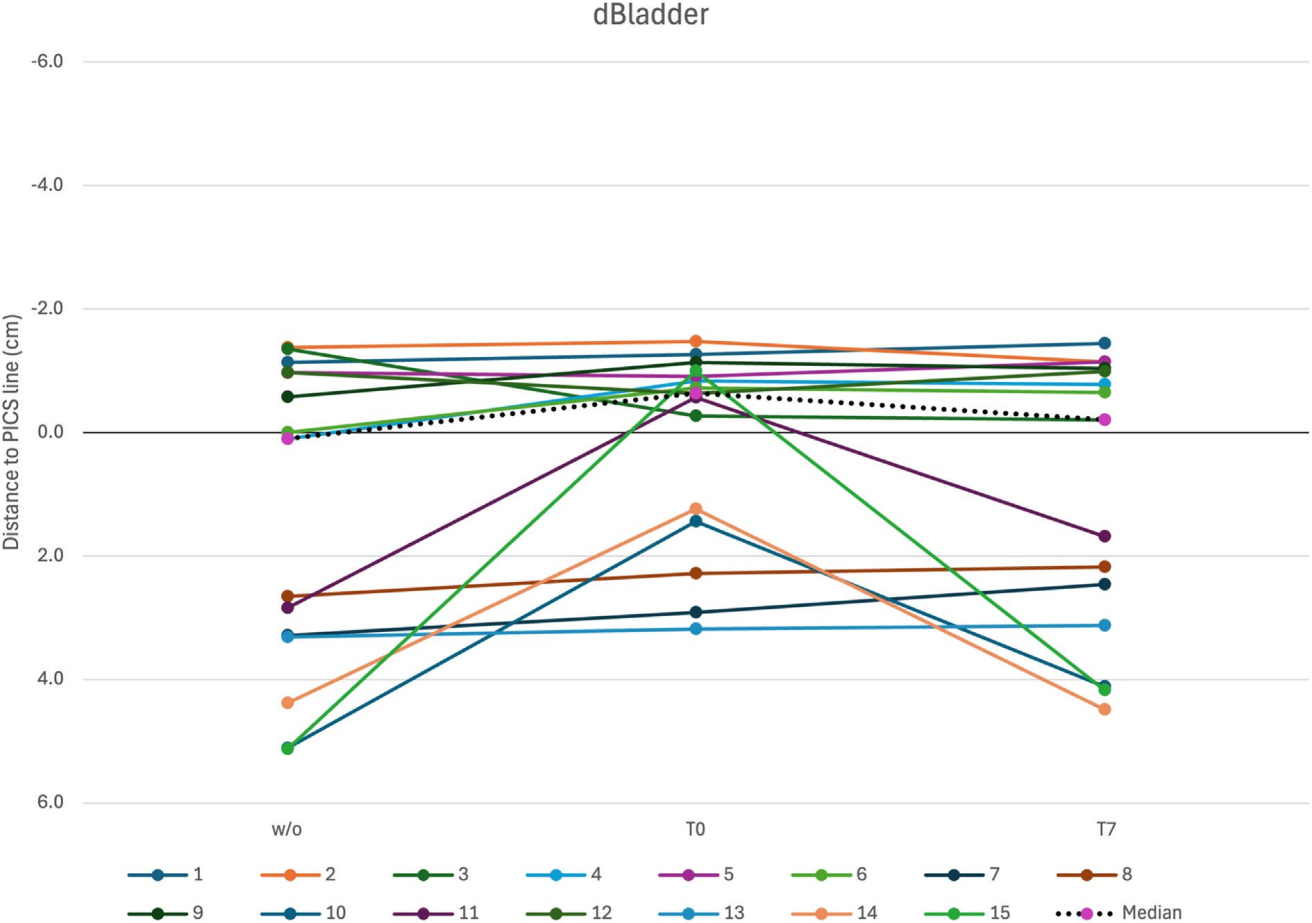
The distance between the lowest point of the bladder and the Pelvic Inclination Correction System (PICS) reference line for the individual patients at the different scan moments. w/o, without pessary; T0, immediately after pessary insertion; T7, 1 week after pessary insertion.

### 3.3 Uterus Position and Orientation

The uterus, measured as the lowest point of the cervix (median (IQR)), was lifted by re‐insertion of the pessary from 0.0 cm (−1.7 cm, 1.3 cm) on the PICS to −2.5 cm (−3.4 cm, −1.5 cm) above the PICS (*p* < 0.001). As shown in Figure 2, no statistically significant change in uterus position was observed 1 week after pessary re‐insertion (−2.1 cm, −3.4 cm, −1.3 cm) above the PICS (*p* = 0.470).

**FIGURE 2:**
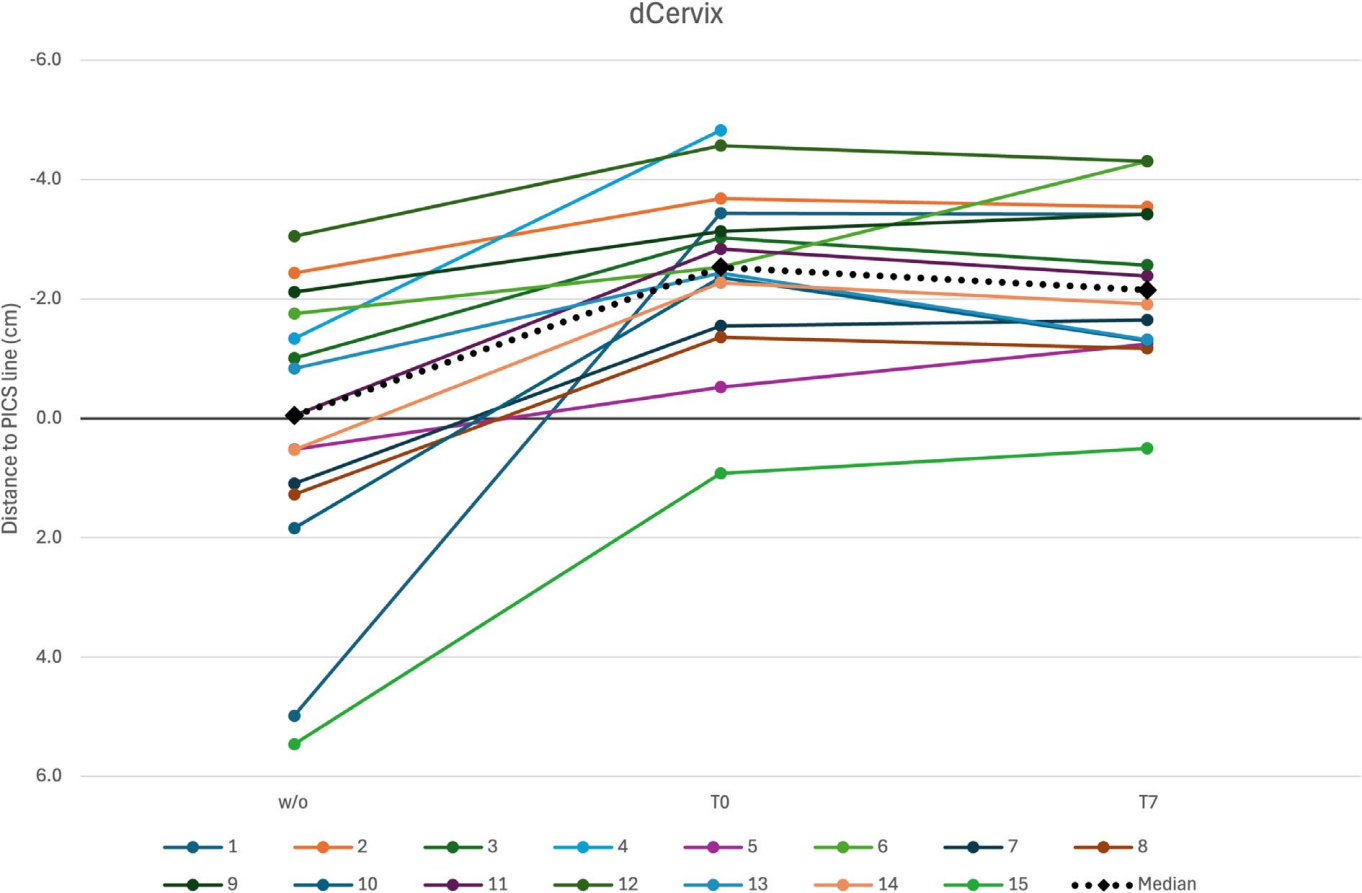
The distance between the lowest point of the uterus (cervix) and the Pelvic Inclination Correction System (PICS) reference line for the individual patients at the different scan moments. w/o, without pessary; T0, immediately after pessary insertion; T7, 1 week after pessary insertion.

In three patients the uterus was retroverted (angle < 60 degrees) before and after pessary insertion. The uterus angle increased from 97 (78, 104) degrees to 115 (100, 149) degrees after pessary re‐insertion (*p* = 0.008), resulting in an anterior rotation of the uterus. This did not change after 1 week of pessary usage (113 (69, 125) degrees, *p* = 0.198). In 13 out of 15 patients (87%) the lowest point of the cervix 1 week after pessary re‐insertion showed a clinically relevant lift of 1 cm or more relative to the position prior to pessary insertion.

### 3.4 Pessary Position and Orientation

The lowest point of the pessary was positioned higher after 1 week of usage from 0.7 cm (−0.3 cm; 1.2 cm) below the PICS line immediately after re‐insertion to 0.3 cm (−0.5 cm; 1.1 cm) below the PICS (*p* = 0.020, Figure 3). The angle of the pessary to the PICS did not change significantly from 56 (43, 61) degrees to 54 (46, 66) degrees (*p* = 0.820).

**FIGURE 3:**
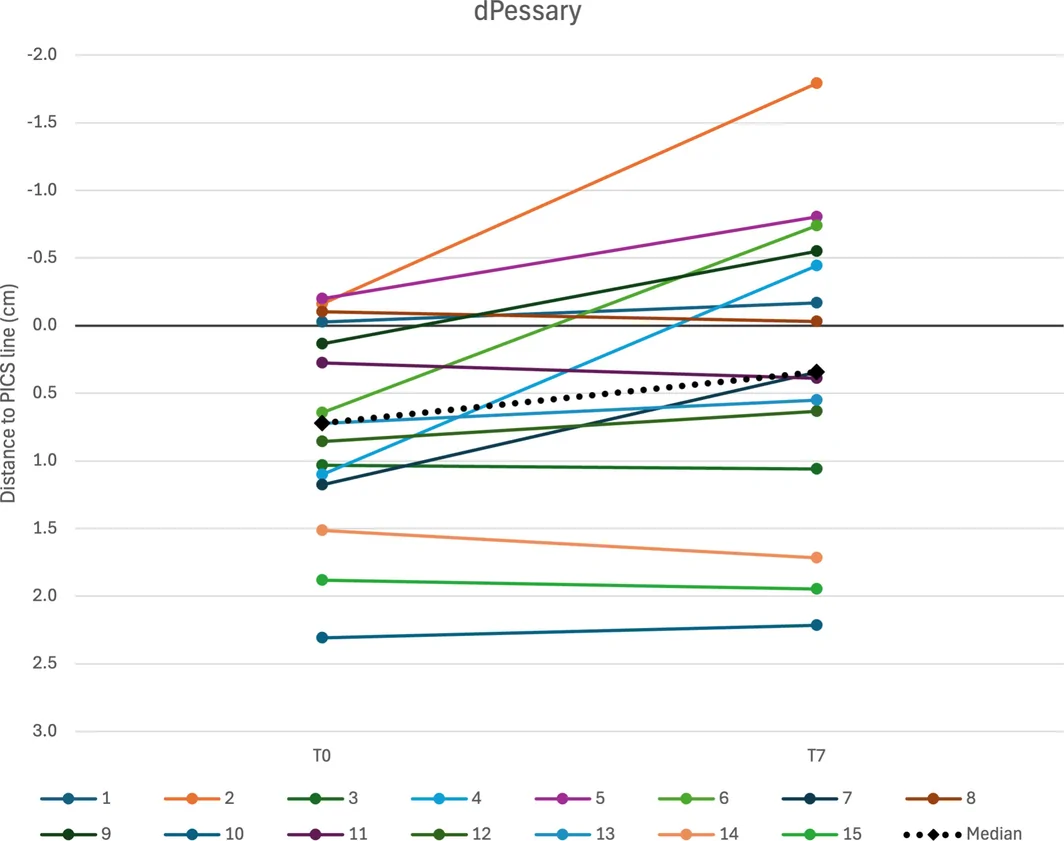
The distance between the pessary and the Pelvic Inclination Correction System (PICS) reference line for the individual patients at the different scan moments. T0, immediately after pessary insertion; T7, 1 week after pessary insertion.

### 3.5 Symptomatic Outcomes

All patients reported POP symptom reduction induced by pessary treatment (Figure S2). More specifically, before pessary treatment 14 out of 15 patients reported bulge symptoms, nine reported pelvic pressure, seven reported defecatory problems and four reported urinary incontinence. During pessary treatment 13 out of 14 patients reported an improvement of the bulge symptoms, seven out of nine of the pelvic pressure, and two out of seven of the defecatory symptoms. None of the patients reported improvement of urinary incontinence, although the answer to this question was missing for one patient. With respect to side effects of pessary use, one patient reported pain and three patients experienced bothersome vaginal discharge.

## 4 Discussion

### 4.1 Main Findings

This study shows that pessary insertion in long term pessary users leads to an initial significant lift of both bladder and cervix. Whereas the uterus position and angulation remained stable in the week after re‐insertion, the lowest point of the bladder descended to a comparable level as before pessary insertion. Considering the symptomatic outcomes, improvement of bulge (13 out of 14 patients) and pressure (seven out of nine patients) complaints was seen, but no improvement in urinary incontinence was observed.

### 4.2 Strengths and Limitations

The most important strength of our study is the use of upright MRI. Women experience their POP symptoms mainly when they are upright in their day‐to‐day routines. Although supine imaging during Valsalva was thought to provide a more accurate representation, previous research showed that upright MRI visualized an even larger POP extent [20]. In addition, when comparing upright MRI to POP‐Q measurements (in supine position) it was shown that POP extent is underestimated with POP‐Q [11]. As women use a pessary to relieve their POP symptoms mainly during the day, assessing the effect of the pessary in an upright position is essential.

Another strength of our study is the addition of an MRI scan 1 week after insertion. In clinicians' experience the organ position and pessary position can be different directly after pessary insertion, and after wearing the pessary for some time. This was confirmed in our study, especially for the bladder position. The pessary position itself did change statistically significant, but with a median of 0.4 cm this may not be considered clinically relevant.

A limitation of our study is the fact that we did not study the posterior compartment. As is known from previous literature [7], and experience in the clinic, posterior compartment prolapse is a risk factor for failure of the pessary to relieve POP symptoms. The posterior compartment is difficult to analyse in our scans, due to the lack of contrast agent in the vagina and rectum, and the lower image quality provided by the 0.25 Tesla MR scanner in combination with an upright position, making it more difficult for some patients to stand completely still. On the other hand, 13 out of 15 patients in our population had predominant prolapse of bladder or uterus, which made the assessment of the posterior compartment of less importance in this group.

Although upright scanning is a great advantage of this study, it also has some limitations. The 0.25 T field strength of our upright MR scanner may be considered a limitation since it hampers the image quality. However, for the purpose of this study, the image quality was sufficient to perform the analyses. Thereby, the use of long acquisition times in MRI (+−5 min per sequence) might be considered another limitation as no organ mobility was visualized. Although dynamic scans, i.e., a single frame per second, are feasible using this scanner enabling visualization of a Valsalva manoeuvre, this is only possible at low resolution images and in 2D. As upright rest scanning was found to be reliable in determining the extent of POP and the pessary position in earlier studies [6, 20], we decided to only scan in rest.

A third limitation of our study is the lack of validated questionnaires. As this was a feasibility study on the anatomical effect of pessary insertion, a non‐validated questionnaire was used, which makes correlating anatomic POP correction to symptom relief difficult. Ideally, for future research, a larger study population is needed that fills out validated questionnaires in order to correlate MRI assessment to clinical findings.

### 4.3 Interpretation

Several previous imaging studies evaluated pessary position and orientation in the pelvis. The studies by Maubon et al. and Komesu et al. [21, 22] specifically focused on bladder neck support in patients with stress urinary incontinence. They concluded that pessary insertion resulted in a superior displacement of the urethra, and support of the bladder neck and bladder base, while in the present study we found only limited and temporary lift of the bladder. Unfortunately, these studies were not performed in POP patients and did not report on POP symptoms and other pelvic anatomy. This makes further comparison to our upright results in POP patients difficult.

Studies evaluating pessary treatment in POP patients have thus far nearly all used supine MRI with the patients performing Valsalva. Boogaard et al. focused on the pessary location and motion of three pessary types in nine patients that continued to use their pessary (fitting pessaries) and six patients in ineffective pessary treatment due to pessary movement or expulsion (non‐fitting pessaries). They concluded that non‐fitting pessaries were located more caudally and rotated more clockwise (smaller pessary‐PICS angle), in comparison to fitting pessaries. In their group, the lowest points of the fitting pessaries were 1.1 cm below the PICS line, and angles to the PICS were 74 degrees and 79 degrees for ring pessaries with and without support. This is a slightly lower and more vertical position than in our upright scans, which may be explained by the difference in scan position. The effect of the pessary on the pelvic organs was not taken into account Boogaard et al. [10]. Another study using MRI identified pelvic anatomic characteristics which were associated with successful pessary fitting, such as total vaginal length and cervical length [23]. However, the effect of pessary insertion on these characteristics was not described, nor the position of the target organs (bladder and uterus), while our results show that the effect of the pessary on the position of the uterus may play a significant role in POP symptom reduction.

This research has several implications in the prediction of treatment success in the clinic. First of all, we see clinical improvement in 14 out of 15 patients, independent of POP extent at baseline. The only woman who reported limited improvement of symptoms was a woman with minimal POP to start with. This could mean that, although some studies have suggested that advanced‐stage POP could be a contra‐indication for pessary treatment [24], a larger apical prolapse is not necessarily a contraindication for pessary treatment. The results in our population show that also in patients with stage 3 POP, pessary treatment can be effective in relieving symptoms. This is also in line with the literature review on this topic by Manzini et al. that showed that advanced‐stage POP is not associated with unsuccessful pessary fitting [25]. Secondly, although our population was too small to draw definitive conclusions, our findings suggest that uterus lift may be the most important factor in relieving POP symptoms such as pressure and bulge feeling. This is in line with previous suggestions in literature that stresses the importance of apical correction in surgical POP repair [26, 27, 28].

The effect of pessary insertion on the bladder however seems minimal both anatomically and when looking at urinary symptoms, especially in women with more severe bladder prolapse. This suggests that pessary treatment is less likely to be successful in women with an isolated anterior compartment prolapse and urinary symptoms as a main complaint.

This exploratory study shows the anatomical effect of pessary insertion in the upright position. However, to draw definitive conclusions on the symptomatic outcomes, research in a larger population using validated questionnaires would be useful. It seems in our population that the symptomatic improvement of urinary symptoms after pessary insertion is limited. Future research should focus on the correlation between symptom improvement and anatomical correction of the prolapse by insertion of a pessary.

Besides, the use of upright MRI in research settings provides additional insights into POP and POP treatment, which can be translated to the clinical situation without the need for upright imaging in a clinical setting.

## 5 Conclusion

Pessary insertion induces a significant initial lift of the bladder and uterus. The change in position and angulation of the uterus is stable over the first week of usage, in contrast to the bladder, where the lowest point appears to redescend near the position before pessary insertion. These differences in bladder and uterus position could imply that, equivalent to surgical POP treatment, especially the uterus lift is important in relieving POP symptoms, in both patients with a predominant anterior and apical prolapse. A more extensive study including a larger study population and a more detailed analysis on clinical outcome is essential to validate these findings.

## Author Contributions

A.v.d.S.: data collection, data analysis, manuscript writing. J.A.S.: data analysis, manuscript writing. M.P.: data collection, manuscript editing. F.F.J.S.: project development, manuscript editing. F.v.d.N.: manuscript editing. A.T.M.G.: project development, manuscript editing.

## Funding

This work was supported by the Reggeborgh Foundation by providing a unique Reggeborgh Research Fellowship for researcher JS. The Reggeborgh Foundation is not the clinical trial sponsor for this research, has no direct interest in patient care or this research, and has no involvement in the research.

## Disclosure

This work was presented at the IUGA 50th Annual meeting June 18–21, 2025 in Barcelona Spain.

## Ethics Statement

The study was approved in October 2020 by the institution's board of directors and medical ethics committee CMO region Arnhem‐Nijmegen, The Netherlands and registered as NL74061.091.20. All patients gave written informed consent. The study was conducted in accordance with the Declaration of Helsinki.

## Consent

All patients gave written informed consent.

## Conflicts of Interest

The authors declare no conflicts of interest.

## Supporting information


**Figure S1:** Landmark annotation. a. Midsagittal MRI view with the Sacrococcygeal Inferior Pubic Point (SCIPP) line (dashed blue), PICS line drawn (blue). The dashed yellow line depicts the perpendicular distance of the lowest point of the bladder to the PICS (d_Bladder_), the dashed pink line the distance of the lowest point of the cervix to the PICS line. The red line depicts the orientation of the endometrial line to the PICS line, resulting in the uterus angle (dashed red). b. Midsagittal view of the same patient with the pessary inserted. The dashed green line depicts the perpendicular distance of the lowest pessary center point to the PICS line. The brown line depicts the orientation of the pessary relative to the PICS line, resulting in the pessary angle (dashed brown).

**Figure S2:** POP complaints before and after pessary insertion.

## Data Availability

The data that support the findings of this study are available on request from the corresponding author. The data are not publicly available due to privacy or ethical restrictions.
