# Supplementary material for: The Effect of Pessary Insertion on Pelvic Anatomy: A Prospective Observational Study Using Upright MRI
Source: BJOG. 2026 Jun 15;133(12):2151–8. doi: 10.1111/1471-0528.70280 (PMC13659428; doi:10.1111/1471-0528.70280)

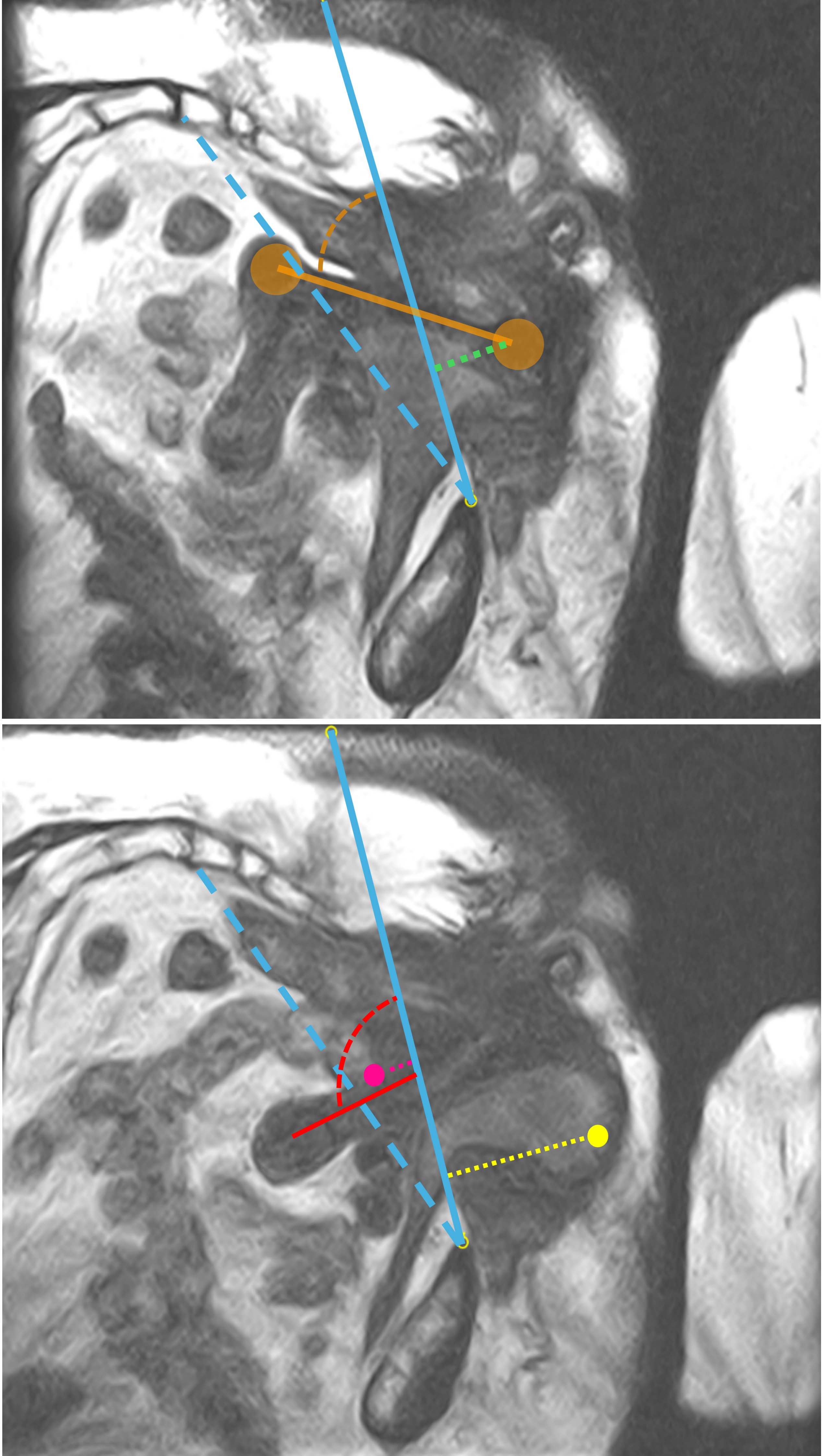

Supplement: Supplementary file 1 [file BJO-133-2151-s001.pdf]
